# Maternal Left Ventricular Function in Uncomplicated Twin Pregnancies: A Speckle-Tracking Imaging Longitudinal Study

**DOI:** 10.3390/jcm11185283

**Published:** 2022-09-07

**Authors:** Rossana Orabona, Edoardo Sciatti, Enrico Vizzardi, Ivano Bonadei, Marco Metra, Enrico Sartori, Tiziana Frusca, Antonio Pinna, Rino Bellocco, Federico Prefumo

**Affiliations:** 1 Maternal Fetal Medicine Unit, Department of Obstetrics and Gynecology, University of Brescia, 25123 Brescia, Italy; 2 Section of Cardiovascular Diseases, Department of Medical and Surgical Specialties, Radiological Sciences and Public Health, University of Brescia, 25123 Brescia, Italy; 3 Department of Obstetrics and Gynecology, University of Parma, 43121 Parma, Italy; 4 Department of Statistics and Quantitative Methods, University of Milano-Bicocca, 20126 Milan, Italy; 5 Department of Medical Epidemiology and Biostatistics, Karolinska Institutet, 171 65 Stockholm, Sweden

**Keywords:** twin, pregnancy, echocardiography, ejection fraction, diastole, systole, 2D strain, speckle-tracking echocardiography, left ventricular, mass, diastolic dysfunction, tissue Doppler imaging

## Abstract

Objective: The knowledge of maternal cardiovascular hemodynamic adaptation in twin pregnancies is incomplete. We aimed to longitudinally investigate maternal left ventricular (LV) function in uncomplicated twin pregnancies. Methods: 30 healthy and uncomplicated twin pregnant women and 30 controls with normal singleton pregnancies were prospectively enrolled to undergo transthoracic echocardiography at 10–15 week’s gestation (w) (T1), 19–26 w (T2) and 30–38 w (T3). LV dimensions and volumes, as well as LV ejection fraction (LVEF), mass (LVM) and diastolic parameters (at transmitral pulsed wave Doppler and mitral annular plane tissue Doppler), were calculated. Speckle-tracking imaging was also applied to evaluate LV global longitudinal (GLS), radial and circumferential 2D strains. Results: During twin pregnancy, maternal LV dimensions, volumes and LVM had an increasing trend from T1 to T3, similar to singletons, while LVEF remained stable. There was LV remodeling/hypertrophy in 50% of women at T2 and T3 in both groups. Diastolic function had a worsening trend from T1 to T3 with no differences between twins and singletons, except for higher LV filling pressure (i.e., E/E′) at T2 in twins. Two-dimensional strains did not vary during gestation in either group, except for a linear trend to increase (i.e., worsen) GLS in singletons. Radial and circumferential 2D strains were impaired in about half of the women at each trimester, while GLS was altered in one-fourth/one-third of them in both groups. Conclusion: Maternal LV geometry, dimensions and function are significantly impaired during twin pregnancies, in particular in the second half of gestation, with no significant differences compared to singletons.

## 1. Introduction

In pregnancy, significant cardiac structural and hemodynamic changes occur. Cardiovascular (CV) remodeling involves the whole heart [1]. Left ventricular (LV) mass (LVM) increases by 30–40% at term above nonpregnant values with a concentric hypertrophy pattern developing in the third trimester [1]. The increase in the LVM plateaus in the third trimester when changes adjust for higher body mass index (BMI) [1]. This dramatic increase outperforms that achieved only by exercise by athletes, who might gain 25% eccentric remodeling [2]. CV physiological changes are required to allow adequate fetal growth and development. For this reason, maternal hemodynamics in women carrying twin pregnancies are thought to show greater modifications than in singletons. Kametas et al. found more pronounced changes in twins than in singletons, including a rise in cardiac output (CO) and LVM and LV ejection fraction (LVEF) [3]. In addition, twin pregnancies are characterized by a higher risk of adverse CV outcomes, e.g., pre-eclampsia [4]. Understanding the normal CV changes in pregnancy is essential to prevent them and also to care for pregnant patients with CV diseases. Furthermore, maternal CV maladaptation may be demonstrated early in pregnancy and used to predict the occurrence of pregnancy complications [5,6,7]. Limited data are available on maternal hemodynamics in twins [3,8,9,10]. Although transthoracic echocardiography is the most common imaging technique used in pregnancy, it has some limitations, including interobserver variability in interpretation and poor image quality in some cases. Using novel diagnostic tools, such as speckle-tracking echocardiography (STE), might positively impact further risk assessment thanks to a deeper investigation of cardiac functioning. STE promises to reduce inter- and intraobserver variability in the assessment of myocardial function and to improve healthcare cost-effectiveness through the early identification of subclinical disease [11,12]. The main purpose of this study was to longitudinally assess maternal cardiac function by means of STE in a cohort of uncomplicated twin pregnant women and to compare it with singleton gestations.

## 2. Materials and Methods

As described in a previous paper on the same population [13], from February 2015 to September 2016, every twin pregnant attending the ultrasound laboratory at the Maternal Fetal Medicine Unit of the Obstetrics and Gynecology Department of the University of Brescia, Italy for the 1st trimester exam were enrolled in a prospective and consecutive way. Increased nuchal translucency, abnormal sonographic findings of either fetus and monoamnionicity represented a priori exclusion criteria. Subjects were scheduled for a combined assessment including fetal sonographic evaluation and maternal cardiologic assessment (i.e., blood pressure measurement and echocardiography) in a stable temperature environment, thrice during pregnancy (T1, 10–15 weeks’ gestation (w); T2, 19–26 w; and T3, 30–38 w). Every woman gave her written informed consent. The study complied with the Declaration of Helsinki, was approved by the local ethics committee and ran following the STROBE indications [14]. Exclusion criteria were: a previous complicated pregnancy (e.g., pre-eclampsia, fetal growth restriction (FGR), intrauterine fetal death or three or more consecutive spontaneous miscarriages); ascertained or suspected fetal anomalies in the present pregnancy; FGR of one or both fetuses (defined as an estimated fetal weight of either fetus < 5th centile, or estimated fetal weight discrepancy greater than 20%); amniotic fluid imbalance or suspected twin-to-twin transfusion syndrome; maternal history of chronic diseases (e.g., hypertensive disorders, diabetes mellitus and renal or immune disorders); traditional CV risk factors (e.g., smoking habit, dyslipidemia and obesity); and drug use. According to the same inclusion criteria, we enrolled healthy subjects with singleton pregnancies attending our unit in the same timespan. Chorionicity was ascertained at the first trimester scan and confirmed by pathology examination after delivery. Demographic and clinical data were collected from obstetrical charts for every woman. The exams were performed by a physician blinded to the women’s data to reduce intra- and interobserver variability.

### 2.1. Blood Pressure Measurement

A standard, calibrated, electronic sphygmomanometer (OMRON Healthcare, Hoofddorp, The Netherlands) was used to measure blood pressure at each arm, at rest in a 45° reclining sitting position. Systolic blood pressure (SBP) was considered elevated if higher than 140 mmHg and diastolic blood pressure (DBP) if higher than 90 mmHg. Two more measurements were taken at the arm with the highest blood pressure, and the average value was calculated. Blood pressure was assessed by the same staff member, at the same part of the day and adopting the same device. Mean arterial pressure (MAP) was defined as (SBP + 2 × DBP)/3.

### 2.2. Traditional and Tissue Doppler Echocardiography

Echocardiographic examinations were performed using a Vivid 7 machine (GE Healthcare, Milwaukee, WI, USA) with a 3.5 MHz transducer. Digital loops were stored on the hard disk of the echocardiograph for on-line and off-line analyses and transferred to an EchoPac, Vingmed workstation (GE Healthcare, Milwaukee, WI, USA) for off-line analysis. Participants were studied in the left lateral decubitus position and images were acquired from standard parasternal and apical windows. LV dimensions, volumes and LVM were obtained according to current guidelines and LVEF calculated by means of Simpson’s biplane method [15]. LVM was obtained by the equation 0.8 × {1.04 × [([LVEDD + IVST + PWT]3 − LVEDD3)]} + 0.6 (g), and relative wall thickness (RWT) was defined as 2 × PWT/LVEDD, LVEDD being the LV end-diastolic diameter, IVST the interventricular septum thickness and PWT the posterior wall thickness at end-diastole [15]. Concentric remodeling was defined by RWT > 0.42 with a normal LVM index, while concentric hypertrophy by RWT > 0.42 and LVM index > 95 g/m^2^ (for females) and eccentric hypertrophy by RWT < 0.42 and LVM index > 95 g/m^2^ (for females) [15]. LV diastolic function was defined according to guidelines, considering transmitral Doppler inflows and tissue Doppler imaging (TDI) at basal segments [16]. Myocardial performance index (MPI) was calculated as (IVCT + IVRT)/ET, IVCT being isovolumic contraction time, IVRT isovolumic relaxation time and ET ejection time at TDI. Valvular alterations were screened for according to guidelines [17,18].

### 2.3. Speckle-Tracking Echocardiography

A two-dimensional (2D) strain calculates myocardial deformation from a 2D point of view. Negative strain means shortening, while positive indicates thickening of a given myocardial segment. STE analysis using a commercially available automated function image technique was applied for the assessment of LV global longitudinal strain (GLS) from apical long-axis slices (long-axis and two- and four-chamber views) [19]. Endocardium was traced at end-systole on the 3 apical views. They were conventionally split in six segments. The operator tracked the speckles frame-by-frame on the six LV segments and manually modified those who failed the procedure until the automatic approval by the software. GLS is defined as the average of the 18-segment longitudinal strain (six per apical view: two-, three- and four-chamber ones). In addition, LV radial and circumferential strains were derived by short-axis view, tracking the endocardium at the papillary muscle level, conventionally split in six segments. We used the reference values for the three strains reported by Kocabay et al. [20]. Figures describing this technique are reported elsewhere [21]. We defined good-quality images if almost 4 segments out of 6 did not require manual interpolation. No patients were excluded from STE analysis.

### 2.4. Statistical Analysis

Continuous variables were reported as mean ± standard deviation (SD) and categorical ones as frequency (*n*) and percentage (%). Student’s *t*-test was run to compare means for continuous variables. The χ^2^ test (or Fisher’s exact test) was adopted to assess differences between proportions.

Every clinical outcome was studied by means of multilevel mixed-effects linear regression analysis. With respect to a standard two-way ANOVA for repeated measurements, such an approach considers each outcome as a linear model with random intercepts and slopes. This assumption fits models with a general covariance matrix depending on time and also uses all nonmissing information on repeated data. The model to be fitted was:

Y_ij = β_0 + β_01 × Twin + β_1 t_j + β_2 t_j^2 + 〖b_i0 + b〗_i1 t_j + e_ij (1)

where:

i is the subject and j the time of the measure.
β_0 is the fixed intercept.
β_01 is the parameter related to the twin dependency.
β_1 and β_2 represent the type of time trend (linear or quadratic).
b_i0 〖+ b〗_i1 t_ij + e_ij is the error term expressed in terms of random intercept, random slope and residuals, where terms b_i0, 〖 b〗_i1 and e_ij have zero mean.

Finally, a quantile regression analysis was run to design boundary curves. This analysis extends the longitudinal mixed model, focusing on the chosen quantile.

To better compare the models, they were corrected for maternal age.

For every outcome, the best-fitted model was selected by means of the minimum Akaike criterion. To discover the best model, an initial linear model hypothesis, adopting the twin pregnancy as covariate, was analyzed and, if needed, its polynomial degree was elevated.

R and R-Studio were utilized to analyze databases and to run traditional statistics (mean, SD, etc.), while longitudinal data analysis was obtained with Stata 14.2 with xtmixed() function, and quantile regression was performed with the lqmm() method from the lqmm R package Version 1.5.8, Marco Geraci, London, UK [22].

For every statistic, *p*-value < 0.05 was considered significant.

## 3. Results

A total of 39 women with uncomplicated twin pregnancies and 34 with healthy singleton ones fitted the inclusion criteria and were enrolled in a prospective way [13]. Among them, 13 women were then excluded (9 twin and 4 singleton), so that 30 women per group reached the end of the study (the causes for exclusion are listed in Table S1). Monochorionic pregnancies were 11 out of 30 twin cases (37%). Three visits (one per trimester) were attended by 24/30 (80%) twins and 28/30 (93%) singletons. In total, 2/30 (7%) women in the twin group missed the T2 visit, while 4/30 (13%) twins and 2/30 (7%) singletons delayed the T3 visit.

Table 1 reports the demographic characteristics of the cohort, as described in a previous paper [13]. Age was higher in women with twin pregnancy (*p* < 0.01), while delivery time earlier and Caesarean section rate greater (*p* < 0.001). Body mass index and body surface area augmented during pregnancy in both groups, with a significant quadratic trend but without intergroup differences.

**Figure 1:**
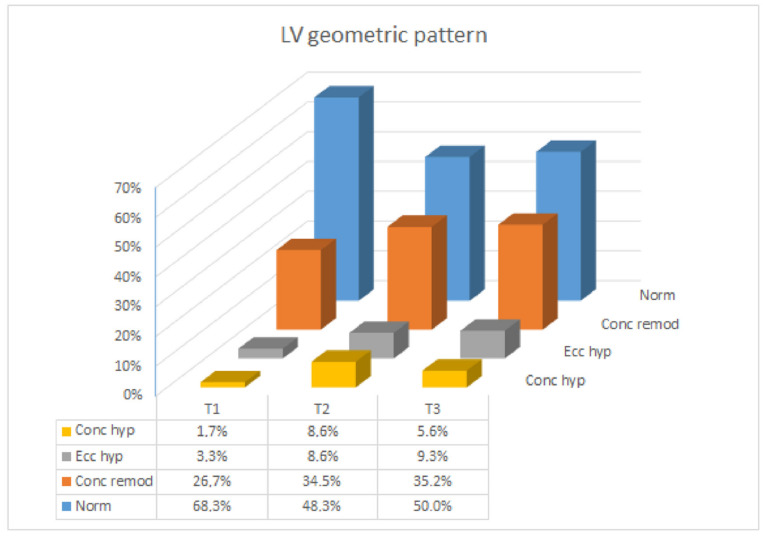
Left ventricular geometric pattern in the whole cohort.

Echocardiographic data are reported in Table 2. LVEF remained stable throughout gestation in both twin and singleton pregnancies without significant intergroup differences (as indicated by longitudinal analysis where no β_01 and β_1 parameters differed significantly from zero). LV end-diastolic volume (EDV) had an increasing trend from T1 to T3 in twins (although this was not significant in the longitudinal model using all data), while end-systolic volume (ESV) increased throughout gestation in both groups as well as IVST. At T3, LVEDD reached values significantly greater in twin pregnancy than in singletons (*p* = 0.04). Consequently, LVM (both indexed for actual BSA or pre-pregnancy BSA) showed a trend to increase from T1 to T3 with no intergroup differences (as confirmed by the longitudinal models). RWT did not vary throughout twin gestation, similar to singleton pregnancy. LV remodeling/hypertrophy was more pronounced at T2 than T1 in both twins and singletons (*p* = 0.018 and *p* = 0.017, respectively), but the LV geometric pattern did not differ between twin and singleton gestations at each time interval. Figure 1 represents the LV geometric pattern during gestation in the whole study cohort. Overall, a normal LV geometric pattern was present in two-thirds of patients at T1, decreasing to 50% at T2 and T3; the rate of LV concentric remodeling increased from one-fourth at T1 to one-third at T2 and T3, and the percentage of LV hypertrophy (both concentric and eccentric) significantly increased from 5% at T1 to 15% at T2 and T3 in twins (*p* = 0.015) (as suggested by the quadratic trend). On the contrary, the concentric pattern (either remodeling or hypertrophy) was similar in the two groups at each trimester.

As concerns diastolic function (Table 2), E wave significantly dropped (quadratic trend) from T1 to T3, while A wave and DT remained stable throughout twin and singleton gestation. Regarding TDI findings, A′, S’ and IVA (either septal or lateral) did not vary during pregnancy (both in twins and singletons). On the contrary, septal and lateral E′ significantly increased in both groups (linear model), except for lateral E′ which was less negative (i.e., worse) at T2 among twins than in singletons. Septal E′/A′ had a significant trend in reduction during singleton pregnancies, while lateral E′/A′ did not show the same trend. E/E′ (representing LV filling pressure) was greater in twins than in singletons at T2 with a significant trend to increase from T1 to T3 in the first group. MPI and IVRT remained stable throughout twin and singleton gestation, while IVCT was greater in twins than in singletons at T3. Grade I diastolic dysfunction was absent at T1, while it regarded one woman (2%) with twin pregnancy at T2 (with LV concentric remodeling) and two women (3%) at T3 (one among twins with normal LV geometric pattern and one among singletons with LV concentric hypertrophy).

Data are given as mean ± SD. *p* (trend) refers to a longitudinal regression run on the subgroup of 30 patients. If reported, the model has *p*-value < 0.05, for details see Table 3.

Data are given as mean ± SD. EDV, end-diastolic volume; ESV, end-systolic volume; LVEF, left ventricular ejection fraction; IVST, interventricular septum thickness; PWT, posterior wall thickness; LVEDD, left ventricular end-diastolic diameter; LVM, left ventricular mass; BSA, body surface area; RWT, relative wall thickness; DT, deceleration time; IVRT, isovolumic relaxation time; IVCT, isovolumic contraction time; ET, ejection time; MPI, myocardial performance index; BMI, body mass index; BSA, body surface area; ESV, end-systolic volume; LVEF, left ventricular ejection fraction; IVST, interventricular septum thickness; PWT, posterior wall thickness; LVM, left ventricular mass; IVCT, isovolumic contraction time.

**Figure 2:**
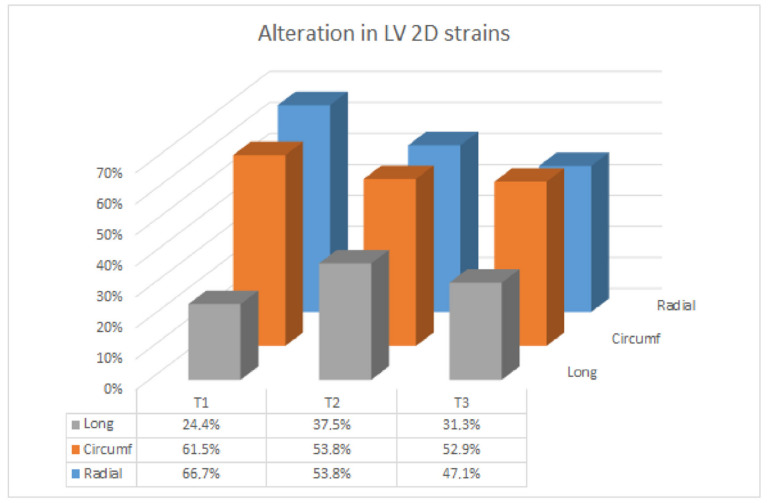
Left ventricular 2D strain alterations in the whole cohort.

STE data (Table 2) were available for 39/60 (65%) women at T1, 39/58 (67%) at T2 and 51/54 (94%) at T3 for radial and circumferential 2D strains and for 41 out of 60 (68%) cases at T1, 40 out of 58 (69%) at T2 and 48 out of 54 (89%) at T3 for GLS. Two-dimensional strains were stable and similar between twin and singleton pregnancies at each trimester, with the exclusion of a linear trend in worsening (i.e., increasing) for GLS in singleton gestation. Radial and circumferential 2D strains were altered in about half of subjects at every time interval, while GLS was impaired in about one-fourth/one-third of cases (Figure 2).

Table 3 highlights the linear regression models considering pregnancy status (twin or singleton) and gestational age as independent variables. None of the LV parameters seemed to be dependent from multiple gestation. On the contrary, several parameters (e.g., LVEF, ESV, IVST, PWT, LVM, E, E/A, septal and lateral E′ and E′/A′, E/E′, ET, radial 2D strain and GLS) depended on gestational age.

## 4. Discussion

The main findings of this study can be summarized as follows: (1) during pregnancy, particularly if multiple, LV volumes, LVM and LV filling pressure increase; (2) some diastolic parameters tend to worsen from T1 to T3 in both twin and singleton pregnancies; and (3) GLS, as well as circumferential and radial strains, did not significant vary during gestation, with no difference between twin and singleton gestation, although 2D strains are altered in a high percentage of women with twin pregnancy.

Pregnancy is characterized by profound hemodynamic adaptation in order to meet the increased metabolic demands of the mother and fetus and to ensure adequate uteroplacental circulation. Indeed, systemic vascular resistance (and blood pressure) falls from the first trimester, with a nadir in the second one, while CO increases till delivery. Consequently, cardiac remodeling occurs to face these changes, principally with increased LVM [1,23].

Limited data are available about maternal hemodynamics during multiple pregnancies. Among these, Kametas et al. found a significantly higher CO among twins than in singletons [3], as did Sima et al., reporting higher stroke volume [24]. More recently, Ghi et al. described a significant worsening in LV systolic (expressed by a decrease in LVEF, fractional shortening and systolic anterograde myocardial velocity (S′)) and diastolic function through twin gestation [25]. We hereby confirmed these data showing an increase in LV volumes, LVM and filling pressures (e.g., E/E′), with a tendency to diastolic dysfunction (shown by a reduced E/A), particularly in twin pregnancies. Moreover, we documented a high prevalence of LV remodeling and hypertrophy in the second half of both twin and singleton pregnancies, thus reflecting the profound maternal hemodynamic changes occurring in normal pregnancy. Modifications in maternal cardiac function according to chorionicity have been recently investigated by our group in a multicenter study [26]. The data showed a significant decrease in CO and a rise in total vascular resistance in MC compared with dichorionic (DC) twins. Furthermore, during MC pregnancies, the impairment of diastolic function seemed to be less pronounced than in DC, presumably due to a lower circulating volume.

In the present paper, we assessed GLS as well as radial and circumferential LV strains, which had never been previously studied in twin pregnancies. Although we found no differences in LV strains among cases and controls, we demonstrated a high rate of LV strain alteration (particularly as concerns radial and circumferential) since T1, in association with LV remodeling and diastolic impairment, remarking that pregnancy is a condition in which the heart faces profound hemodynamic challenges. STE has been applied to overcome the limitations of conventional echocardiography. It is angle-independent, not greatly influenced by preload or afterload and not affected by heart movements [11,12]. Studying longitudinal, circumferential and radial deformations, 2D strain gives a more comprehensive evaluation of LV systolic function, from both regional and global points of view, focusing on subendocardial fibers, which are the first to be damaged in CV disorders. GLS has better prognostic value for predicting major adverse CV events than does LVEF [27], and it is highly reproducible [28]; it could even provide additional information when LVEF is normal or almost normal [29,30].

Outside of pregnancy, there is a well-established trend for basing treatment on hemodynamic profiling. For example, in the NICE guidance on adult hypertension [31], treatment is refined by reference to age and ethnicity, as these are associated with distinct underlying hemodynamic pathophysiologies: older and black patients generally have a vasoconstrictive state while young patients tend to have a hyperdynamic state. In pregnancy, hemodynamic states change rapidly both as a normal part of pregnancy adaptation and pathologically in complicated pregnancies. Hemodynamic monitoring in twin gestations is therefore likely to be required frequently in order to maintain and refine treatment regimens in specific conditions more frequent among twins (e.g., hypertensive disorders of pregnancy). Although there is little research in pregnancy regarding the feasibility and efficacy of using maternal hemodynamics to inform individualized monitoring and treatment, previous reports highlighted the use of serial hemodynamic monitoring in pregnancy to guide treatment of hypertension because it significantly reduces the rate of severe hypertension itself [32]. In this study, we wanted to assess maternal hemodynamic performance status in uncomplicated twin pregnancies in order to contribute to guiding clinicians in the management of complicated twins, which is essential to caring for patients, particularly those with CV diseases, with the aim of reducing the rate of nonresponse to treatment, identifying women who require dose adjustment or those who progress rapidly to severe forms of CV disorders requiring treatment in the emergency department, and thereby lowering the rate of severe forms of the diseases.

The main strengths of this present paper lie in its prospective study design with a longitudinal assessment of maternal cardiac function from the first to the third trimester of gestation. Moreover, a whole evaluation of maternal LV function by means of STE had never been previously carried out in twin pregnancies. A detailed assessment of maternal hemodynamics in twins might offer a better insight to the process of maternal adaptation to twin pregnancy in order to also improve the understanding of the pathophysiology of CV complications occurring more frequently during twin gestations [33].

We acknowledge the following limitations to the study. Firstly, we cannot provide accurate reference ranges for each variable due to the small number of enrolled women. Secondly, the absence of a post-delivery evaluation does allow us to show which variables eventually normalize and when they do so. Thirdly, as already discussed, a significant proportion of monochorionic twins was enrolled; considering that CO is marginally higher in dichorionic ones [26], this can somewhat explain why we did not demonstrate a significant difference between twin and singleton pregnancies.

## 5. Conclusions

To conclude, pregnancy is a condition of high hemodynamic load and determines profound changes in the left ventricle. STE is able to detect a high percentage of alterations, which are the expression of the damage induced by hypertrophy and remodeling. In how many women it persists remains to be clarified. No substantial differences were found in multiple pregnancies. However, our data are limited by the small sample size. Larger studies with a postpartum assessment are needed to extend our knowledge.

## Figures and Tables

**Table 1:** Demographical characteristics of the enrolled cohort.

Characteristic	Twin Pregnancy (*n* = 30)	Singleton Pregnancy (*n* = 30)	*p*
Age at delivery (years)	34.5 ± 4.3	31.5 ± 4.0	0.01
Body mass index (kg/m^2^):			
Pre-pregnancy	22.2 ± 3.4	23.0 ± 3.6	0.35
1st trimester	22.7 ± 3.2	23.7 ± 3.8	0.30
2nd trimester	24.5 ± 3.3	25.0 ± 3.7	0.54
3rd trimester	27.4 ± 2.9	27.0 ± 4.0	0.69
*p*	<0.00009 (*)	<0.00009 (*)	
Body surface area (m^2^):			
Pre-pregnancy	1.7 ± 0.2	1.7 ± 0.2	0.50
1st trimester	1.7 ± 0.2	1.7 ± 0.2	0.52
2nd trimester	1.8 ± 0.2	1.7 ± 0.2	0.30
3rd trimester	1.9 ± 0.2	1.8 ± 0.2	0.04
*p*	<0.00009 (*)	<0.00009 (*)	
Parity: - Nulliparous - Primiparous - Multiparous	20 (67) 10 (33) -	21 (70) 7 (23) 2 (7)	0.28
Gestational age at delivery (weeks)	35^+3^ ± 2^+2^	39^+2^ ± 2^+4^	<0.001
Chorionicity: - Dichorionic - Monochorionic	19 (63) 11 (37)	- -	-
Caesarean section	26 (87)	8 (27)	<0.001

Data are given as mean ± SD or *n* (%). (*) The trend has expression: $Y_{j}=\beta_{0}+\beta_{1}t+{\;\beta }_{2}t_{j}^{2}$.

**Table 2:** Left ventricular findings obtained by conventional and speckle-tracking echocardiography at each trimester in twin versus singleton pregnancies.

	1st	2nd	3rd	*p* (Trend)		Longitudinal Model	Twin Dependency
EDV (mL)	90 ± 21	98 ± 21	103 ± 22	0.0140	Twin	no	
	93 ± 20	103 ± 22	99 ± 21	0.2829	Singleton		
	0.4894	0.4055	0.5351		*p*		
ESV (mL)	36 ± 11	39 ± 11	44 ± 13	0.0147	Twin	yes, linear	no
	36 ± 9	41 ± 12	41 ± 11	0.0399	Singleton	Y=36.28+3.11t	
	0.8033	0.5179	0.4615		*p*		
LVEF (%)	60 ± 6	61 ± 7	58 ± 7	0.1992	Twin	yes, linear	no
	61 ± 6	61 ± 6	59 ± 7	0.1491	Singleton	Y=61.18+0.77t	
	0.4923	0.9945	0.5646		*p*		
IVST (mm)	8.6 ± 1.5	9.2 ± 1.3	9.7 ± 1.7	0.0104	Twin	yes, linear	no
	9.0 ± 1.5	9.5 ± 1.8	10.2 ± 1.9	0.0035	Singleton	Y=8.78+0.58t	
	0.2621	0.4874	0.2904		*p*		
PWT (mm)	8.6 ± 1.5	9.3 ± 1.5	9.0 ± 1.4	0.0954	Twin	yes, quadratic	no
	8.4 ± 1.6	9.4 ± 1.8	9.3 ± 1.4	0.0006	Singleton	Y=8.48+ − 1.39 t$\;-\;0.52{\;t}^{2}$	
	0.6756	0.7961	0.4429		*p*		
EDD (mm)	44.0 ± 4.0	46.0 ± 4.1	47.0 ± 6.5	0.0232	Twin	no	
	44.1 ± 5.7	44.7 ± 5.2	43.7 ± 5.1	0.7175	Singleton		
	0.9791	0.2917	0.04201		*p*		
LV mass (g)	121 ± 29	143 ± 22	152 ± 39	0.0001	Twin	yes, quadratic	yes
	123 ± 24	142 ± 38	142 ± 31	0.0010	Singleton	Y=122.02 + + 29.04 t $\;-\;8.48{\;t}^{2}$	
	0.8189	0.8566	0.3032		*p*		
LV mass/BSA (g/m^2^)	71 ± 18	81 ± 13	81 ± 20	0.0061	Twin	yes, quadratic	yes
	72 ± 13	81 ± 18	79 ± 15	0.0166	Singleton	Y=71.51 + − 15.05 t$\;-\;5.44{\;t}^{2}$	
	0.7068	0.9316	0.7495		*p*		
LV mass/BSA pre-pregnancy (g/m^2^)	72 ± 19	84 ± 15	89 ± 23	0.0002	Twin	yes, quadratic	yes
	73 ± 13	84 ± 20	85 ± 17	0.0010	Singleton	Y=72.39 + − 17.00 t$\;-\;4.96{\;t}^{2}$	
	0.7209	0.9741	0.4449		*p*		
RWT	0.39 ± 0.08	0.41 ± 0.09	0.39 ± 0.11	0.8455	Twin		
	0.39 ± 0.11	0.43 ± 0.11	0.43 ± 0.11	0.0580	Singleton	no	
	0.9776	0.48	0.171		*p*		
E (m/s)	0.85 ± 0.13	0.90 ± 0.18	0.76 ± 0.17	0.0022	Twin	yes, quadratic	yes
	0.89 ± 0.15	0.89 ± 0.14	0.80 ± 0.17	0.0013	Singleton	Y=−0.87+ − 0.08 t$\;-\;0.061{\;t}^{2}$	
	0.2931	0.7717	0.351		*p*		
A (m/s)	0.54 ± 0.13	0.56 ± 0.10	0.53 ± 0.14	0.5392	Twin	no	
	0.54 ± 0.10	0.55 ± 0.10	0.53 ± 0.14	0.8510	Singleton		
	0.9087	0.6333	0.7064		*p*		
E/A	1.66 ± 0.45	1.64 ± 0.42	1.47 ± 0.26	0.0417	Twin	yes, linear	no
	1.72 ± 0.43	1.64 ± 0.32	1.54 ± 0.43	0.0685	Singleton	Y=1.69 − 0.08t	
	0.6186	0.9298	0.4737		*p*		
DT (ms)	169 ± 19	170 ± 18	165 ± 17	0.4874	Twin	no	
	170 ± 22	174 ± 19	178 ± 35	0.1952	Singleton		
	0.8466	0.3331	0.09913		*p*		
E′ septal (m/s)	−0.143 ± 0.033	−0.145 ± 0.035	−0.122 ± 0.034	0.0049	Twin	yes, quadratic	yes
	−0.154 ± 0.037	−0.159 ± 0.033	−0.133 ± 0.035	0.0003	Singleton	Y=−0.14 + − 0.016 t$\;+\;0.013{\;t}^{2}$	
	0.2254	0.1188	0.2537		*p*		
A′ septal (m/s)	−0.088 ± 0.031	−0.092 ± 0.025	−0.084 ± 0.033	0.4296	Twin	no	
	−0.092 ± 0.022	−0.095 ± 0.028	−0.091 ± 0.041	NA	Singleton		
	0.5572	0.6119	0.4818		*p*		
S’ septal (m/s)	0.102 ± 0.017	0.105 ± 0.017	0.097 ± 0.022	0.5325	Twin	no	
	0.101 ± 0.021	0.109 ± 0.021	0.106 ± 0.032	0.4597	Singleton		
	0.902	0.4434	0.2004		*p*		
E′/A′ septal	1.56 ± 0.41	1.61 ± 0.59	1.43 ± 0.51	0.2799	Twin	yes, linear	no
	1.78 ± 0.51	1.57 ± 0.75	1.45 ± 0.56	0.0178	Singleton	Y=1.68 − 0.11 t	
	0.07091	0.7864	0.9087		*p*		
E′ lateral (m/s)	−0.199 ± 0.042	−0.182 ± 0.047	−0.153 ± 0.043	<0.0009	Twin	yes, linear	no
	−0.204 ± 0.042	−0.207 ± 0.039	−0.172 ± 0.036	0.0001	Singleton	Y=−0.20 + 0.018 t	
	0.6402	0.03022	0.08381		*p*		
A′ lateral (m/s)	−0.088 ± 0.031	−0.092 ± 0.025	−0.084 ± 0.033	0.6003	Twin	no	
	−0.092 ± 0.022	−0.095 ± 0.028	−0.091 ± 0.041	NA	Singleton		
	0.5572	0.6119	0.4818		*p*		
S’ lateral (m/s)	0.118 ± 0.031	0.118 ± 0.027	0.108 ± 0.032	0.1967	Twin	no	
	0.124 ± 0.030	0.128 ± 0.039	0.121 ± 0.035	0.7420	Singleton		
	0.4448	0.2375	0.1484		*p*		
E′/A′ lateral	2.48 ± 1.01	2.13 ± 0.86	2.01 ± 0.75	0.0904	Twin	yes, linear	no
	2.29 ± 0.61	2.31 ± 0.65	2.10 ± 0.68	0.1692	Singleton	Y=2.39 + 0.16 t	
	0.3842	0.3807	0.6418		*p*		
E/E′	5.2 ± 1.42	5.7 ± 1.3	5.7 ± 1.3	0.0417	Twin	yes, linear	no
	4.9 ± 1.3	4.9 ± 1.1	5.5 ± 1.5	0.0940	Singleton	Y=5.05 + 0.28 t	
	0.4138	0.02152	0.5064		*p*		
IVRT (ms)	48 ± 15	47 ± 18	52 ± 19	0.2586	Twin	no	
	47 ± 13	45 ± 13	47 ± 14	0.9702	Singleton		
	0.8597	0.763	0.2798		*p*		
IVCT (ms)	71 ± 21	62 ± 19	67 ± 17	0.3373	Twin	yes, linear	no
	68 ± 19	68 ± 21	57 ± 14	0.0069	Singleton	Y=69.60 − 3.92 t	
	0.4794	0.5417	0.02106		*p*		
ET (ms)	294 ± 29	287 ± 25	283 ± 29	0.1447	Twin	no	
	290 ± 24	284 ± 19	271 ± 31	0.0050	Singleton		
	0.6327	0.6328	0.1575		*p*		
MPI	0.41 ± 0.11	0.39 ± 0.10	0.42 ± 0.09	0.6933	Twin	no	
	0.40 ± 0.10	0.40 ± 0.11	0.39 ± 0.09	0.6783	Singleton		
	0.6657	0.4259	0.157		*p*		
Radial 2D strain (%)	21.5 ± 15.0	25.2 ± 13.4	25.4 ± 13.5	0.4542	Twin	yes, linear	no
	15.8 ± 17.2	22.8 ± 27.3	27.6 ± 22.1	0.0599	Singleton	Y=19.74 − 3.69 t	
	0.2732	0.7207	0.6653		*p*		
Circumferential 2D strain (%)	−14.2 ± 6.0	−15.7 ± 3.8	−15.3 ± 5.0	0.5792	Twin	no	
	−14.7 ± 3.4	−13.7 ± 3.3	−15.3 ± 5.0	0.8121	Singleton		
	0.7554	0.08604	0.8539		*p*		
Global longitudinal 2D strain (%)	−20.2 ± 1.8	−20.3 ± 2.7	−19.5 ± 2.1	0.1357	Twin	yes, linear	no
	−20.4 ± 2.4	−19.0 ± 2.4	−19.4 ± 2.1	0.0238	Singleton	Y=−20.31 − 0.51 t	
	0.6886	0.1303	0.9097		*p*		

**Table 3:** Longitudinal regression analysis model based on: Y_ij = β_0 + β_01*Twin + β_i1 t_ij + β_2 t_ij^2 + b_(0i) + b_i1 t_ij + e_ij.

	Base Value		β (Twin)		β (Time)		β (Time^2^)		Model
	Coeff.	*p*	Coeff.	*p*	Coeff.	*p*	Coeff.	*p*	*p*
BMI	22.60 ± 0.46	0.00			0.15 ± 0.13	0.26	0.45 ± 0.04	<0.0009	<0.00009
BSA	1.69 ± 0.02	0.00			0.006 ± 0.004	0.123	0.013 ± 0.01	<0.0009	<0.00009
VTS	36.28913 ± 1.373047	0.00			3.115208 ± 0.9573086	0.001			0.0011
LVEF	61.18302 ± 0.7782806	0.00			−1.099469 ± 0.5498972	0.046			0.0456
IVST	8.780679 ± 0.1854537	0.00			0.582123 ± 0.14959	<0.0009			
PWT	8.483333 ± 0.1983069	0.00			1.388778 ± 0.4009307	0.001	−0.5246509 ± 0.1943994	0.007	0.0002
LV mass	122.0206 ± 3.616209	0.00			29.04015 ± 7.675927	<0.0009	−8.480164 ± 3.6985	0.022	<0.00009
LV mass/BSA	71.51432 ± 2.07294	0.00			15.0495 ± 4.112765	<0.0009	−5.443507 ± 1.975872	0.006	0.0001
LV mass/BSA pre-pregnancy	72.3954 ± 2.167393	0.00			17.00408 ± 4.409177	<0.0009	−4.965437 ± 0.0207455	0.019	<0.00009
E	0.8745057 ± 0.017099	0.00			0800678 ± 0.0428861	0.062	−0.0616711 ± 0.005970	0.003	<0.00009
E/A	1.699659 ± 0.054071	0.00			−0.0838877 ± 0.0304139	0.006			0.0058
E′ septal	−0.1480847 ± 0.0044928	0.00			−0.0169843 ± 0.0086277	0.049	0.0133066 ± 0.0041706	0.001	<0.00009
E′/A′ septal	1.678654 ± 0.0632461	0.00			−0.1165775 ± 0.0468856	0.013			0.0129
E′ lateral	−0.2048665 ± 0.0051701	0.00			0.017748 ± 0.002872	<0.0009			<0.00009
E′/A′ lateral	2.387295 ± 0.1003394	0.00			−0.1638165 ± 0.0687277	0.017			0.0171
E/E′	5.04785 ± 0.1625199	0.00			0.2579962 ± 0.1013529	0.011			0.0109
IVCT	69.59747 ± 2.553735	0.00			−3.923314 ± 1.558401	0.012			0.0118
Radial 2D strain	19.74516 ± 2.705705	0.00			3.694701 ± 1.820146	0.042			0.0424
Global longitudinal 2D strain	−20.30775 ± 0.3287788	0.00			0.5085934 ± 0.1919979	0.008			0.0081

## Data Availability

Data available upon request.

## Supplementary Materials

The following supporting information can be downloaded at: https://www.mdpi.com/article/10.3390/jcm11185283/s1, Table S1: Reasons to leave the study. The last visit attended is reported in brackets.
