# Supplementary material for: Maternal Left Ventricular Function in Uncomplicated Twin Pregnancies: A Speckle-Tracking Imaging Longitudinal Study
Source: J Clin Med. 2022 Sep 7;11(18):5283. doi: 10.3390/jcm11185283 (PMC9504023; doi:10.3390/jcm11185283)
Supplement: Supplementary file 1 [file jcm-11-05283-s001.zip › jcm-1884509-supplementary.pdf]

Table S1. Reasons to leave the study. The last visit attended is reported in brackets.

**Twin Pregnancy (*n* = 9)**	**Singleton Pregnancy (*n* = 4)**
3 fetal malformation/chromosomopathy (T1, T1, T1)<br>2 fetal loss (T1, T2)<br>1 HELLP syndrome and fetal loss (T2)<br>3 lost at follow-up (T1, T1, T1)	1 pre-eclampsia and IUGR (T2)<br>3 lost at follow-up (T1, T1)

HELLP, hemolysis elevated liver enzymes low platelet; IUGR, intrauterine growth restriction. Data are given as mean ± SD or n (%). The trend has the expression: $Y_j = \beta_0 + 〖\beta_1 t+ \beta〗_2 t_j^2$.
